# Streptococcal Toxic Shock Masking Lemierre's Syndrome

**DOI:** 10.7759/cureus.93627

**Published:** 2025-10-01

**Authors:** Aye Oo, Matthew Murphy, Marina Morgan

**Affiliations:** 1 Infectious Disease, University Hospitals Birmingham NHS Foundation Trust, Birmingham, GBR; 2 Radiology, Royal Devon University Healthcare NHS Foundation Trust, Exeter, GBR; 3 Microbiology and Infection, Royal Devon University Healthcare NHS Foundation Trust, Exeter, GBR

**Keywords:** fusobacter necrophorum, group a beta hemolytic streptococcus, internal jugular venous thrombosis, intravenous immunoglobulin, lemierre's syndrome, streptococcal toxic shock syndrome

## Abstract

We report a case of a previously healthy woman presenting with septic shock unresponsive to seven litres of intravenous fluid resuscitation, broad-spectrum antimicrobials, necessitating admission to intensive care. The history and clinical presentation suggested streptococcal toxic shock syndrome (STSS), and group A beta-hemolytic streptococcus (GAS) was isolated from blood cultures. Persisting leg weakness, neck and spinal pain led to an MRI, which showed an inflammatory process on the right side of the neck, confirmed by CT angiogram as right internal jugular venous thrombosis. The triad of cervical lymphadenopathy, internal jugular venous thrombosis and sepsis is a classical feature of Lemierre's syndrome, but here caused by a rarely associated pathogen, namely GAS. Our case illustrates the necessity for investigating all possible foci of invasive GAS (iGAS) and repeating a careful clinical examination if not improving.

## Introduction

Lemierre’s syndrome (also known as necrobacillosis) is named after Andre Lemierre, who in 1936 finally identified the causative agent of ‘anaerobic post anginal sepsis’ as a Gram-negative anaerobic bacillus now known as Fusobacterium necrophorum. Patients typically present with fever, neck pain and swelling after pharyngitis, and rapidly progress to pneumonia with cavitating lesions and sepsis. Lemierre’s classical description involved 20 cases of invasive infection and septic thrombophlebitis following acute pharyngotonsillitis, of whom 18 died [1]. Septic embolization from internal jugular vein thrombosis resulted in metastatic infection, particularly to the lung and large joints [1].

The incidence of Lemierre’s syndrome varies with age, being commonest in adolescence and young adults. A Danish study found the overall incidence of Lemierre’s syndrome to be 3.6 cases per million population per year, with the highest incidence (14.4 per million/year) in 15-24-year-olds, and rarely seen in those over 40 years of age [2]. In a series of 712 patients with Lemierre’s syndrome, 58% of positive cultures yielded fusobacteria [3], but this may be an underestimate since fusobacteria can be difficult to culture.

The pathophysiology of infection is uncertain since fusobacteria can be part of the normal oropharyngeal flora. With many cases reported post-viral infection, including infectious mononucleosis, it has been suggested that a breach of the inflamed mucosa allows bacteria to invade the soft tissues of the neck. The pro-coagulopathic fusobacteria then invade the lateral pharyngeal space, where, with close proximity to the vascular bundle, thrombosis can result.

In our case of Lemierre's syndrome, only group A beta-hemolytic streptococcus (GAS) was isolated. Clues from the history and presentation of our case pointed to the early clinical diagnosis and management of streptococcal toxic shock syndrome (STSS). We illustrate the overlap of features of STSS [4] with those of fusobacterial sepsis, outline similarities and differences between the organisms, and compare management strategies for each.

## Case presentation

A previously fit female in her fifties presented in septic shock, after being confined to bed for three days with a recurrent sore throat, massive cervical lymphadenopathy, vomiting and diarrhoea. Her severe generalised myalgia, back and neck pain had not been helped with ibuprofen, and necessitated administration of intravenous morphine. Conjunctival suffusion and symmetrical lower limb weakness were noted, although on examination there was no focal neurology.

Her temperature was 39.7°C, with a tachycardia of 110/min, blood pressure of 73/32 mm Hg, respiratory rate 20 breaths per minute and oxygen saturation 90% on room air. She received our standard empirical “sepsis” regimen of intravenous amoxycillin, gentamicin and metronidazole. Her C-reactive protein was 421 mg/L, with a thrombocytopenia of 59 x 109/L, haemoglobin 9.5 g/dl, creatinine 161 μmol/L and serum albumin 23 g/L. Her peripheral leucocyte count was in the ‘normal’ range, 8.3 x 10^9^/L, but with a lymphopenia of 0.2 x 10^9^/L (Table 1).

**Table 1:** Initial admission laboratory data

Laboratory marker	Units	Patient values	Reference range
White blood cell count	cells/µL	8.3 x 10^3^	4-11 x 10^3^
C-reactive protein	mg/L	421	0-10
Hemoglobin	g/L	95	120-160
Platelets	cells/µL	59 x 10^3^	140-400 x 10^3^
Lymphocytes	cells/µL	0.2 x 10^3^	1.5-4.0 x 10^3^
Creatinine	µmol/L	161	44-80
Albumin	g/L	23	35-50

A chest X-ray demonstrated right lower lobe consolidation. Remaining fluid unresponsive after six litres of fluid, she was admitted to the Intensive Care Unit, where antimicrobials were changed following review by microbiology to cover likely invasive GAS. Antibiotics were changed to intravenous piperacillin-tazobactam and linezolid. Linezolid was chosen because of a worrying trend of increasing GAS resistance to clindamycin in our population - currently almost 40%.

**Figure 1:**
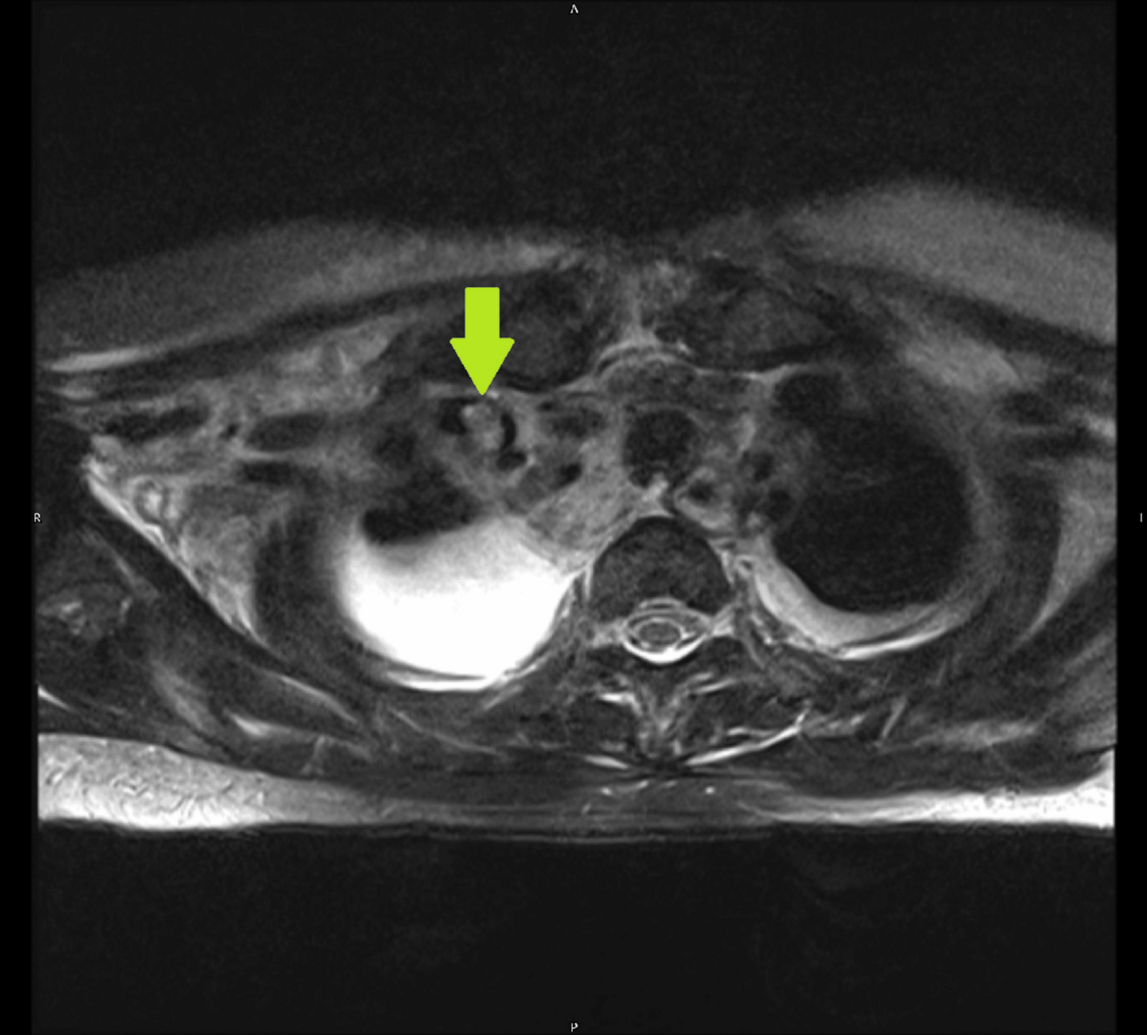
MRI T2 axial image showing thrombus at confluence of right internal jugular vein (IJV) and subclavian vein (arrow).

The next day, with spinal pain necessitating increasing doses of morphine, a spinal MRI was performed to exclude discitis. Oedema of the back muscles, bilateral pleural effusions and inflammatory changes on the right side of the neck and thrombus at confluence of right IJV and subclavian vein that the radiologist reported as “consistent with Lemierre’s syndrome” were noted (Figure 1).

**Figure 2:**
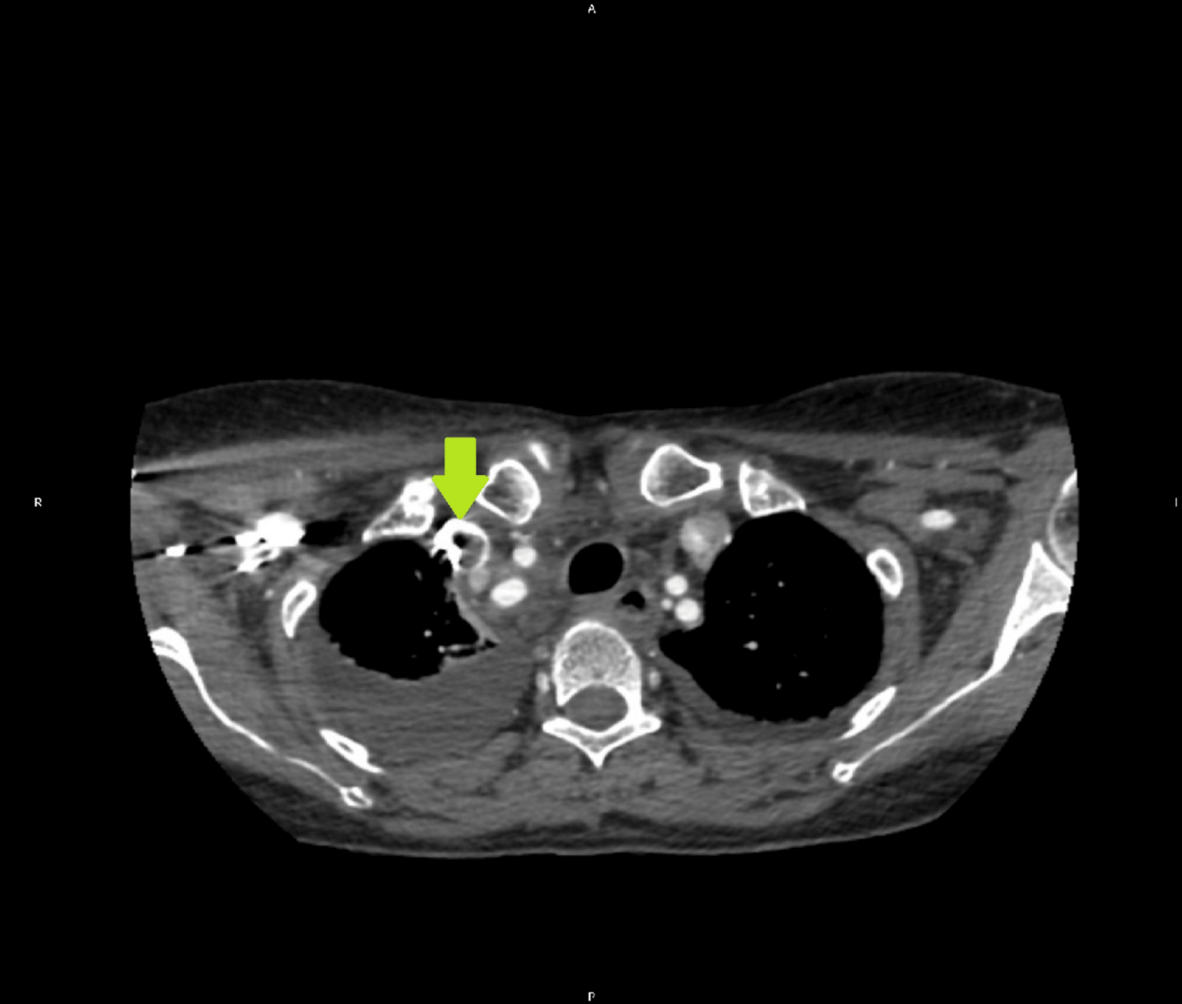
CT angiogram confirming the presence of intraluminal thrombus (arrow).

**Figure 3:**
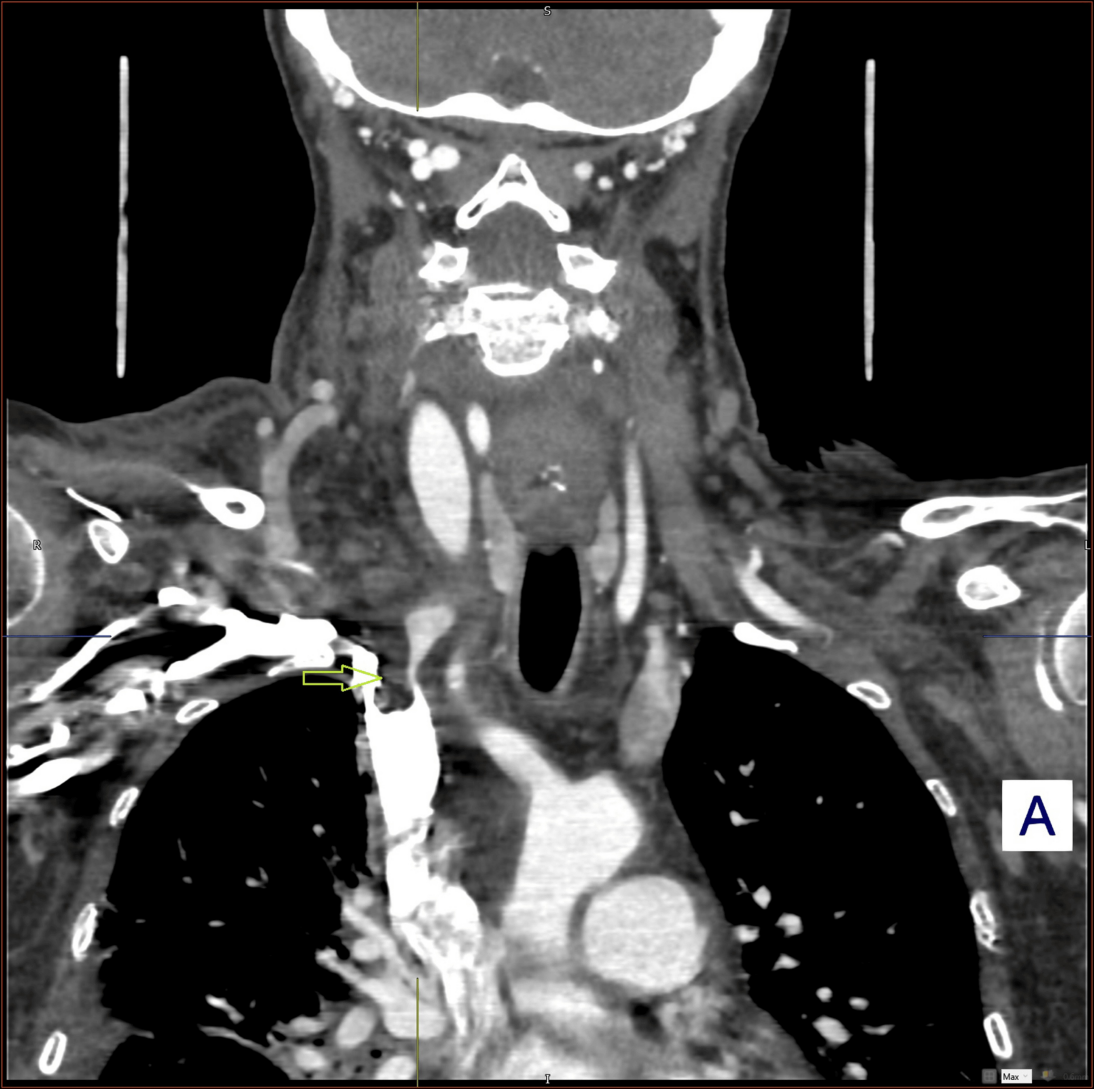
Coronal CT angiography scan showing thrombus in the right internal jugular vein (arrow).

Subsequent CT angiography confirmed the presence of a right internal jugular venous thrombosis (Figures 2, 3).

Piperacillin-tazobactam was replaced with ceftriaxone in order to ensure brain penetration in case of septic embolization of a GAS. With no fusobacteria yet isolated, ceftriaxone and linezolid together provided reasonable anaerobic cover.

Eighteen hours after admission, blood cultures revealed Gram-positive cocci in chains, later confirmed as GAS, sensitive to penicillin, clindamycin and linezolid. Despite intensive efforts after the discovery of the right internal jugular (RIJ) thrombus, no fusobacteria were isolated from any culture. In retrospect, it would have been helpful to have had more than the one blood culture and a throat swab taken before commencing any antimicrobials to maximise the chances of isolating fusobacteria. With reported successes of intravenous immunoglobulin (IVIG) therapy neutralising circulating exotoxins in severe invasive GAS infection unresponsive to conventional treatments [5], IVIG was considered, but there was concern about the possible prothrombotic action of IVIG, and she had begun to respond. By day 5, her peripheral blood parameters began to normalise, with significant clinical improvement. The case for anti-coagulation was debated, but not commenced until day 11 when platelet counts had recovered.

Intravenous antimicrobials continued for 14 days, with intercurrent spikes of temperature, continuing arthralgia and a grumbling CRP attributed to pleural effusions. On day 9, she spiked a fever of 38.4 °C with pain and effusions in both knees and her left wrist, raising the possibility of reactive arthritis. After rheumatology review on day 10, with a CRP level of 119 mg/dl, aspiration and washouts of both knees revealed frank pus with no organisms seen on Gram staining. Cultures remained sterile. Wrist aspiration was not performed.

A transesophageal echo revealed no vegetations. On day 16, oral clindamycin (450 mg six hourly) was commenced. On day 28, she was discharged with resolving right basal lung consolidation and pleural effusion. She completed a total of six weeks total antimicrobial therapy with significant improvement in both knees and left wrist pain. Complete dissolution of the thrombus was demonstrated on follow-up CT pulmonary angiogram six months later.

## Discussion

The presentation of sepsis, sore throat, cervical lymphadenopathy, conjunctival suffusion, diarrhoea and vomiting suggested STSS.

Adult Lemierre’s syndrome due solely to GAS is rare; the three cases reported to date involved elderly patients [6-8]. Those over 45 years of age are more than twice as likely to have other aerobic pathogens causing infection compared with fusobacteria [3].

The commoner aerobes causing Lemierre's syndrome are streptococci of the Streptococcus anginosus group (SAG) [9], with a few cases reported due to Staphylococcus aureus, including MRSA [10,11], and single cases of Gram negatives such as Eikenella [12] and Klebsiella [13] reported. Occasionally, co-infections occur [14].

Fusobacteria and GAS both colonise the oropharynx. Having reached the soft tissues of the neck, both can spread via lymphatics and the blood stream to the lungs. Classical presentations of Lemierre’s syndrome include sore throat (33%), neck mass (23%) and pain (20%) [15], bone and joint pain, gastrointestinal symptoms, and limb weakness [1] - all of which our patient demonstrated. In fusobacterial Lemierre’s, nodular lung infiltrates with cavitation are common, and may progress rapidly in spite of antibiotic treatment. Our patient with GAS Lemierre’s had pleural effusions and lung consolidation, probably resulting from septic emboli. The mortality of Lemierre’s syndrome has decreased significantly since 1936, when 18 of 20 patients died [1]. Mortality has now fallen to 4% [16].

In our case, the diagnosis of Lemierre’s syndrome was made more difficult by the predominant clinical features of STSS and the assumption that severe pharyngitis and cervical lymphadenopathy caused the neck pain. Lemierre's syndrome may easily have been missed completely were it not for continuing spinal pain and leg weakness on day 2 and the initial MRI findings. Our case mirrors the classical presentation of Lemierre’s initial 1936 series, where “The appearance and repetition several days after the onset of a sore throat…. of several pyrexial attacks with an initial rigor, or still more certainly the occurrence of pulmonary infarcts and arthritic manifestations, constitute a syndrome so characteristic that mistake is almost impossible” [1]. Septic arthritis is still a notable feature of Lemierre’s syndrome due to Fusobacterium, present in 12-27% of cases [17], but is uncommon in STSS. Our case of GAS Lemierre’s syndrome probably had bilateral GAS septic arthritis of the knees, sterile pus resulting from antecedent antimicrobials.

The management of Lemierre’s syndrome is primarily medical, using antimicrobials targeting the most likely causative organism, historically Fusobacteria. There are no guidelines for the choice or duration of antibiotic therapy for Lemierre’s. Hence, we suggest consideration of linezolid, meropenem and metronidazole pending identification of the causative organism(s) since this covers all reported organisms implicated to date, has an excellent anaerobic spectrum and penetrates the cerebrospinal fluid. A six-week course of antimicrobials has been recommended to maximise penetration of infected fibrin clot [13]. Shorter courses, especially if combined with interventional radiological removal of clot, surgical drainage or excision of thrombosed veins, may be successful. Surgery may be considered when medical management fails, clot extension occurs, or necrotic material needs removal [10, 13].

In addition to beta-lactams, the empirical antimicrobial management of toxic shock increasingly involves the addition of antimicrobials such as clindamycin or linezolid that lessen exotoxin production [4].

The similarities and differences between Fusobacterium necrophorum-related Lemierre's syndrome and STSS are summarised in Table 2.

**Table 2:** Comparison between Fusobacterium necrophorum-related Lemierre’s syndrome and STSS.

	Fusobacterium necrophorum sepsis, Lemierre's syndrome	Streptococcus pyogenes sepsis, streptococcal toxic shock syndrome
Gram-staining characteristics of organism	Gram-negative bacilli	Gram-positive cocci in chains
Pathogenic factors produced by organism	Endotoxin, Adhesins, Hemagglutinins, Leukocidin, Hemolysin	No endotoxins. Exotoxins acting as superantigens: Streptolysin O, Collagenases, Hemolysin
Carriage	Oropharyngeal carriage, most common in late adolescents, young adults	Oropharyngeal carriage, most common in young children
Site of origin	Oropharynx	Oropharynx. Skin and soft tissue infection
Gastroenteritis	Rare	Common due to enterotoxin action of exotoxins
Generalised body rash, conjunctival suffusion	No	30-40%
Septic emboli	Common lungs, less common joints	Rare
Internal jugular venous thrombosis	A defining characteristic of Lemierre's syndrome	Rare
Antimicrobials	Susceptible to penicillins, metronidazole and clindamycin.	Susceptible to penicillin, usually susceptible to clindamycin. Resistant to metronidazole
Intravenous immunoglobulin	Not indicated. Produces only one exotoxin, a leukotoxin	May be indicated to neutralise several circulating exotoxins if all other measures failing
Mortality	4%	35%

With no clear guidelines available, the role of anticoagulation remains controversial, although over half of patients receive it [18]. Anticoagulation may prevent propagation of thrombus and reduce the risk of septic embolism, although fragmentation and spread of infected clot might be increased. Anticoagulation is recommended for patients failing antimicrobial therapy, those with an underlying thrombophilia, progression of thrombosis, or cavernous sinus thrombosis [18]. Anticoagulation may increase the local activity of antimicrobials [19]. In our patient, anticoagulation was commenced only when the platelet count returned to normal.

## Conclusions

Early imaging of the neck in septic patients with persistent pain or neurological symptoms is vital in all cases of sepsis following oropharyngeal infection. Clinical features of STSS may mask and overlap with those of fusobacterial sepsis. In patients over 45 years of age, Fusobacter is less likely to be the cause of Lemierre’s syndrome. Hence, in older patients with sepsis, neck pain and GAS, Lemierre’s should be actively excluded.

Pending identification of the causal pathogen(s), we suggest meropenem, linezolid and metronidazole as an empirical antimicrobial regimen to cover all bacteria implicated in Lemierre’s to date, and with excellent brain penetration. Antimicrobial therapy should usually be continued for six weeks. The potential benefits and risks of anti-coagulation should be carefully evaluated on an individual basis.

## Appendices

Patient perspective

I first started to feel unwell 10 days prior to my hospital admission. I felt tired, had a sore throat and my glands and under jaw lymph nodes were sore. I assumed it was a virus and took paracetamol.

Five days before my hospital admission, I had an aching back and legs. I felt weak and had little appetite.

The day before my hospital admission I went to bed with a high temperature, chills and sweats. My body was aching. I felt as though I had flu. My neck was stiff, and I had no appetite.

During that night my condition deteriorated rapidly. I became very confused, incontinent and sick. My back was very painful, and I was unable to sit or stand up. My husband called for an ambulance. The paramedics arrived but did not take me to hospital. After checking me they left. A few hours later my husband called them again as I was deteriorating. The ambulance arrived, and I remember that they took a long time ‘stabilizing’ me in the ambulance before driving to the Emergency Dept.

My admission to hospital was a bit of a blur. They initially believed that I had Norovirus.

Things started to happen when I was visited by the microbiologist who explained the diagnosis was probably streptococcal infection. She told me she had secured a bed in the ICU. I remember being taken for a scan, not feeling anxious, just relieved that I was being cared for.

Once in ICU, I was semi aware of my surroundings. I remember having a cannula put in my neck vein which was scary, and various drips being set up. I felt safe.

I have no recollection of being in pain but subsequently understood that I was being given morphine. Hence the strange dreams. I remember the anxious look on the faces of the family.

Once I moved to a hospital ward I was regularly visited by a number of consultants. My legs were very swollen. I was told that I was going to have my knees drained as there was a risk of septic arthritis. I think that this was the first time Sepsis was actually mentioned.

I was very anxious about this diagnosis.

I was totally immobile in bed and required a hoist to access a chair and the commode. I continued to suffer from severe back pain. It was 2 weeks before I could be taken in a wheelchair for a shower. I was being very closely monitored by the medical staff and still required additional oxygen. I also knew that I had been given a ‘serious cocktail of antibiotics’ and that they were working, as my condition was improving. 

Gradually, as I became more mobile, I began to feel more positive about my recovery. I was still not fully aware of the severity of my illness or the full diagnosis.

I knew that my blood pressure and oxygen levels had to increase and my CRP count decrease before I could be discharged. I was having regular visits from the physiotherapist, who taught me how to me to raise myself from the bed and start to stand, unsupported.

When I was discharged, it was in a wheelchair. I had a prescription for further antibiotics and understood that I would then need to take Warfarin for 6 months. This came as a bit of a shock and the significance of my diagnosis, which I by then understood to be Lemierre’s Syndrome with Group A Streptococcal toxic shock, finally sank in.

My physical recovery took 12 months. I continued to experience back pain and had lost limb and core strength. I took up a part-time post with less responsibility and focussed on making a full physical recovery and have subsequently followed a regular yoga and sea swimming (even in winter) exercise regime. I am now very fit, and active.
